# A hydrophobic patch surrounding Trp154 in human neuroserpin controls the helix F dynamics with implications in inhibition and aggregation

**DOI:** 10.1038/srep42987

**Published:** 2017-02-23

**Authors:** Mohammad Farhan Ali, Abhinav Kaushik, Charu Kapil, Dinesh Gupta, Mohamad Aman Jairajpuri

**Affiliations:** 1 Protein Conformation and Enzymology Lab, Department of Biosciences, Jamia Millia Islamia (A Central University), New Delhi-110025, India; 2 Translational Bioinformatics Group, International Centre for Genetic Engineering and Biotechnology (ICGEB), Aruna Asaf Ali Marg, New Delhi-110067, India

## Abstract

Neuroserpin (NS) mediated inhibition of tissue-type plasminogen activator (tPA) is important for brain development, synapse formation and memory. Aberrations in helix F and β-sheet A movement during inhibition can directly lead to epilepsy or dementia. Conserved W154 residue in a hydrophobic patch between helix F and β-sheet A is ideally placed to control their movement during inhibition. Molecular Dynamics (MD) simulation on wild type (WT) NS and its two variants (W154A and W154P) demonstrated partial deformation in helix F and conformational differences in strands 1A and 2A only in W154P. A fluorescence and Circular Dichroism (CD) analysis with purified W154 variants revealed a significant red-shift and an increase in α-helical content in W154P as compared to W154A and WT NS. Kinetics of tPA inhibition showed a decline in association rates (k_a_) for W154A as compared to WT NS with indication of complex formation. Appearance of cleaved without complex formation in W154P indicates that the variant acts as substrate due to conformational misfolding around helix F. Both the variants however showed increased rate of aggregation as compared to WT NS. The hydrophobic patch identified in this study may have importance in helix F dynamics of NS.

Human Neuroserpin (NS) a member of serine protease inhibitor (serpin) superfamily is expressed throughout the nervous system but more specifically at the later stages of neuronal cell development^1^,^2^,^3^. NS inhibits serine protease tissue-type plasminogen activator (tPA), which has been reported to play critical role in memory, synaptic plasticity and brain development^2^,^4^,^5^. Lack of NS activity or its polymerisation can directly lead to dementia and epilepsy^6^,^7^. NS with specific point mutations like S49P, S52R, H338R, G392E and G392R have been recognized to cause familial encephalopathy with neuroserpin inclusion bodies (FENIB)^6^,^7^,^8^,^9^,^10^. These mutations are located around the shutter region and are believed to cause opening of β-sheet A which leads to the formation of polymers via loop-sheet polymerisation mechanism^8^,^9^,^11^. Deficiency in other inhibitory serpins like α_1_-antitrypsin (AAT), α_1_-antichymotrypsin (AAC), C1-inhibitor, plasminogen activator inhibitor-1 (PAI-1) and antithrombin (AT) may result in pathological disorders like emphysema/cirrhosis, chronic obstructive bronchitis, angio-edema, bleeding disorder and thrombosis respectively^12^,^13^,^14^,^15^,^16^. These pathological disorders are collectively termed as ‘serpinopathies’^9^. Under normal conditions the circulatory inhibitory form of wild type (WT) serpin exists in a metastable conformation^17^,^18^,^19^. However, during its inhibitory action on serine proteases it acquires a stable conformation upon cleavage of the reactive center loop (RCL) and insertion into the β-sheet A as an additional strand 4A^20^. During this process the protease remains covalently trapped, almost irreversibly as an acyl enzyme intermediate^3^. As a result the conformational dynamics of serpin inhibition mechanism makes it prone to polymer formation. The latter is attributed to the large scale movements in RCL, helix F and strands of β-sheet A^21^,^22^.

Earlier, a Molecular Dynamics (MD) simulation study has reported the distinct movement of helix F away from β-sheet A during inhibition mechanism, and helix flexibility was found to be of importance during the final stages of NS mediated inhibitory mechanism^23^. Besides, *Gettins PG 2002*^24^ also predicted the movement of helix F during insertion of strand 4A, followed by locking the stable acyl-protease-inhibitor complex after complete insertion. Analysis of AAT variants by *Cabrita et al*.^25^ clearly showed melting and restructuring of helix F at both N and C-terminal end at the later stages of loop insertion during inhibition mechanism. Further, protein engineering based experiments have elucidated that certain mutations in the helix F accelerates polymerisation mechanism of AAT^26^. Intriguingly these predictions were contrasted by detection of an inactive late stage intermediate in the crystal structure of L55P from AAC. The structure revealed that the loop connecting helix F to strand 3A of β-sheet A partially fills the space between the sheet A^27^, leading to loss of its activity. In view of these facts, it is likely that NS inhibition of tPA would also require the movement of the helix F, however the residues contributing to this movement and their exact role in the mechanism that links inhibition and polymerisation still remains unclear.

A conserved W154 residue in helix F is part of a large hydrophobic patch with a network of hydrophobic interactions^28^ with residues of helix F and β-sheet A (Fig. 1a). The patch contain residues Y150, I151, W154 and V155 from helix F, A135 of strand 1A, A109 and F113 from strand 2A and I181 from strand 3A of the β-sheet A. Residues of the hydrophobic patch are conserved in the orthologs of NS and the serpin family (Supplementary Fig. S1). Moreover, a structural superposition of WT (PDB ID: 3FGQ)^29^ and cleaved loop inserted NS (PDB ID: 3F02)^30^ conformation indicated that the loop insertion causes more shift at the bottom of helix F, than at the top, along with the bottom of strands 1A, 2A and 3A of β-sheet A indicating a coordinated movement between the two regions (Fig. 1b). The structural superposition also showed conformational shifts in the loops connecting helix F to the strand 3A at its C-terminal end and strand 1A at the N-terminal end of the cleaved NS. Therefore, in the present study we performed site-directed mutagenesis on NS by mutating W154 to alanine (W154A) and proline (W154P) to investigate the effect of loss of interactions between helix F and β-sheet A on conformation, activity and dynamics of NS with implication on its inhibition and aggregation. The W154A and W154P variants were designed with the premise that strands 1A, 2A and 3A of β-sheet A interacting with W154 are involved in shifting of these strands may increase the polymerisation propensity if interactions with helix F are lost. On the other hand, any change that restricts the helix F movement will impede the insertion of RCL as strand 4A during inhibition. The results of the present study demonstrated that hydrophobic interactions between W154 of helix F and β-sheet A are important and needs to be thoroughly tested for helix F movement during loop insertion and in preventing aggregate formation during inhibition.

## Results

### Molecular Dynamics Simulation Studies

The initial coordinates were taken from PDB ID: 3FGQ^29^ for MD studies and were first assigned the missing atom by using Swiss-Model^31^. The rationale for using it was primarily its high resolution (2.09 Å) as compared to PDBID: 3F5N (3.15 Å)^30^. The study was initiated by performing MD simulations for WT, W154A and W154P NS variants (Supplementary Fig. S2). The Root Mean Square Fluctuation (RMSF) analysis showed a marked increase in fluctuation in strand 1A and decreased mobility in strand 2A in both the variants. Of note the differential fluctuation was found to be lowest in the strand 3A in both the variants as compared to the WT NS. We observed that W154P variant demonstrated greater mobility in helix F, loop and strand 1A region; however, the mobility was reduced in strand 2A than the W154A variant (Fig. 2). In contrast, for W154A, we observed significantly reduced mobility in the helix F region; however helix F at N-terminal of the W154P variant showed comparatively higher mobility. Interface between strands 3C and 4C also showed significantly higher mobility in W154P than W154A. In addition, the superposition of the simulated structures of W154P and W154A highlighted that helix F at the N-terminal end of W154P was deformed, but not in the case of W154A variant (Fig. 3a). The loop connecting strand 1A with helix F was also observed to be shifted, indicating the significant movement of Y150 away from the hydrophobic patch. A structural superposition of the modeled structure of W154P and cleaved NS also demonstrated the partial loosening of helix F at the C-terminal end as compared to cleaved NS structure and also moving away of the loop connecting helix F with strand 3A of the β-sheet A (Fig. 3b). Both the loop connecting helix F to strand 3A and 1A were found to be shifted in case of W154P variant (but not in W154A and WT NS) thereby, resulting in an increased conformational flexibility (Figs 2 and 3). Therefore, it is more likely that hydrophobic patch around W154 might be contributing to helix deformation and formation of an intermediate type of structure where the strands of β-sheet A were still in the native like conformation (Fig. 3b). Based on these *in silico* analysis, we hypothesized that the presence of this hydrophobic patch may contribute to the helix F dynamics in inhibition mechanism of NS.

### Expression and purification

To test our hypothesis based upon *in silico* analysis, site-directed mutagenesis was performed to obtain the W154A and W154P variants using pET28b vector containing WT NS gene encoded with N-terminal poly-His-tag and the amplified variants (Fig. 4a,b) were assessed by DNA sequencing of the complete gene (Supplementary Fig. S3). The variants and the WT NS were transformed into *E. coli* BL21/DE3 cells for expression and purification. The WT NS and 154 variants were purified by two-step chromatography using Ni-NTA Sepharose column and HiLoad 16/60 Superdex 200 column. The variant migrated corresponding to its molecular weight with the same mobility as WT NS (Fig. 4c,d and e). Lanes 5 and 6 showing Ni-NTA elution at 300 mM and 600 mM imidazole were further concentrated, buffer exchanged and loaded onto a Gel-filteration (GF) column for isolating active fraction. Active NS peak eluting at 79 ml was concentrated and buffer exchanged for further analysis. Concentration was determined using molar extinction coefficient of the protein at 280 nm. Only 0.5–1.0 mg of monomer W154P and W154A protein from 4 liter of culture was obtained as compared to a yield of 8.0–9.0 mg of WT NS. These observations point towards a decrease in the expression levels of variants and/or slight conversion to oligomeric forms. Eluted fractions run on 12% SDS-PAGE were confirmed to be homogenous.

### Conformation of WT and mutant proteins

To assess the conformational differences between WT NS and the 154 variants, their fluorometric and CD analysis were conducted and the results are summarized in Fig. 5. Fluorescence emission spectra of W154A and W154P showed a reduction in the emission intensity as compared to WT NS (Fig. 5a). A polymeric NS showed a clear red-shift with characteristic decrease in the emission intensity^32^, matching with spectra of the chemically denatured WT NS^33^. Besides, W154P also revealed a red-shift although the emission intensities of both the variants were comparable. Further, the W154P also showed an increase in the surface hydrophobicity in the presence of 4,4′-Bis-1-anilino naphthalene 8-sulfonate (bis-ANS) as compared to W154A and WT NS indicating that the surface environment of W154P and W154A were possibly different (Fig. 5b). Far-UV CD spectra of the proteins showed a marginal increase in α-helical content of the W154P variant as compared to WT NS and W154A variant (Fig. 5c), indicating a more structured protein, agreeing with the previous study^34^.

### Inhibitory activity of WT and mutant proteins

To obtain insight into the functional role of mutant, inhibition assay was performed to assess inhibitory activity WT NS and the W154 variants. Different concentrations of inhibitor (NS and the W154 variants) were incubated with a constant amount of tPA along with its chromogenic substrate. Progress of the reaction (tPA inhibition) was followed by hydrolysis of substrate with time. Association rate constant (k_a_) was calculated using equation (2) (materials and methods) and is presented in Fig. 6. The experiments were performed under pseudo-first order rate conditions^8^,^35^. W154P revealed a low association rate constant (0.09 × 10^3^ M^−1^s^−1^) as compared to W154A (0.54 × 10^4^ M^−1^s^−1^) and WT NS (3.19 × 10^4^ M^−1^s^−1^), agreeing with the rates shown previously using single chain t-PA (sct-PA)^2^,^36^. The rate of inhibition of W154P variant was significantly reduced, a 6-fold decline in the k_a_ of W154A NS variant was also observed.

To investigate the nature of stable-complex formation between tPA and NS, both were incubated for varying time and assessed on 12% SDS-PAGE (Fig. 7). We observed covalent complex formation in WT NS (Fig. 7a) and W154A (Fig. 7b) but not in case of W154P (Fig. 7c), moreover the cleaved NS was detected indicating a purely substrate behavior with no covalent complex formation (Fig. 7d,e).

### Propensity of polymerisation

Since, NS has also been shown to be less stable that can polymerise upon mild thermal stress^8^,^34^,^37^. Therefore, WT NS and 154 variants were incubated at 45 °C and monitored for the polymer formation on non-denaturating PAGE after taking the aliquots of reaction at different time intervals (Fig. 8). Although, slight conversion to large and small molecular weight polymer were observed, however the major portion of native was not able to form polymer even after 240 min of incubation (Fig. 8a). In contrast, almost all of W154A were converted to short and long chain oligomers just after 15 min of incubation (Fig. 8b). W154P showed conversion to high molecular weight aggregate within 45 min. The W154P reaction solution became turbid after 15 min of incubation at 45 °C, which might be due to the precipitation of the unfolded amorphous material due to partial unfolding of the protein, consequently resulting in unfolded aggregates with absence of oligomeric polymers (Fig. 8c). This might be attributed to the loss of interaction between helix F and strands of β-sheet A.

## Discussion

**Figure 1:**
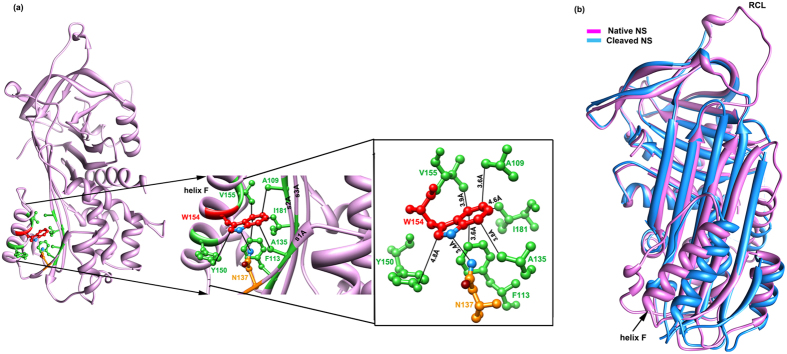
*In silico* analyses of hydrophobic patch in NS. (**a**) Interactions of W154 present on helix F in WT NS. Green residues are involved in hydrophobic interaction while orange residue makes hydrogen bond with W154. (**b**) Superimposition of native PDB ID: 3FGQ^29^ (pink) and cleaved NS PDB ID: 3F02^30^ (blue). The images were generated using Chimera^47^ and the distance was calculated through the program module.

The increased propensity of NS to form polymer was proposed to be due to the labile nature of helix F and its role during inhibition mechanism^25^. The dynamics of helix F after initial docking of tPA to NS through Michaelis complex and during the transition of RCL as strand 4A and the terminal kinetically trapped acyl-intermediate steps still remains poorly understood^38^. A Fluorescence Resonance Energy Transfer (FRET) based analysis had shown two distinct stages of RCL insertion in AAT, describing late stage changes at the interface of helix F and β-sheet A and predicted that energy stored in helix F contributes to the insertion process^39^. We have identified a previously unknown conserved hydrophobic patch at the interface between helix F and β-sheet A centered around W154 which can influence helix F conformational transition during inhibition mechanism. Residues of the hydrophobic patch originating from strand 1A (A135 and N137), strand 2A (A109 and F113) and strand 3A (I181) interacts with the W154 of helix F (Fig. 1a). W154 also interacts with N-terminal (Y150 and I151) and C-terminal (with V155) helix F residues, which may contribute towards helix dynamics. Moreover, the N-terminal and C-terminal of helix F connects the strand 1A and strand 3A respectively (of β-sheet A) through loops. Helix deformation can control the dynamics of the loop to influence the shift in the strands. The strand can shift only if the loops are relaxed possibly due to deformation of the helix F during inhibition mechanism. Previous work demonstrating the increase in polymerisation of AAT due to C-terminal end deformation of helix F revealed that increase in flexibility of the loop is important for inhibition^26^.

We made W154A and W154P variants by site-directed mutagenesis, purified them to homogeneity, and evaluated them for structure-function using WT NS as control. The decrease in fluorescence intensity was observed in both the variants with W154P showing a red-shift indicating partial unfolding and a more hydrophilic environment (Fig. 5a). Further, the rise in surface hydrophobicity and an increased α-helical content was observed in W154P (Fig. 5b,c) but not in W154A and WT NS. Althogether these observations indicate that overall α-helical content of the W154P was higher in contrast to the simulation studies that pointed towards a deformation of the helix F. These results support that both the variants have different conformations. The variants were also denatured in the presence of varying concentration of guanidinium chloride (GdmCl) to assess if helix F has a role in folding mechanism, monitored by intrinsic fluorescence of Trp residues. A two-step unfolding transition was seen in W154A and WT NS at 0.9 M and 2.5 M GdmCl concentration agreeing with prior observation showing denaturation curve of NS^34^,^40^. It was observed that an intermediate formed between 1.5–1.8 M GdmCl was missing in the denaturation profile of W154P but not in W154A and WT NS (Supplementary Fig. S4), resulting in a faster two-step transition. Curiously, W154 contributes in the folding mechanism of NS only when helix F is conformationally deformed (W154P). These results are in accordance with other helix F variants in serpins that have been shown to cause destabilization and that helix F is part of a folding intermediate^25^.

**Figure 2:**
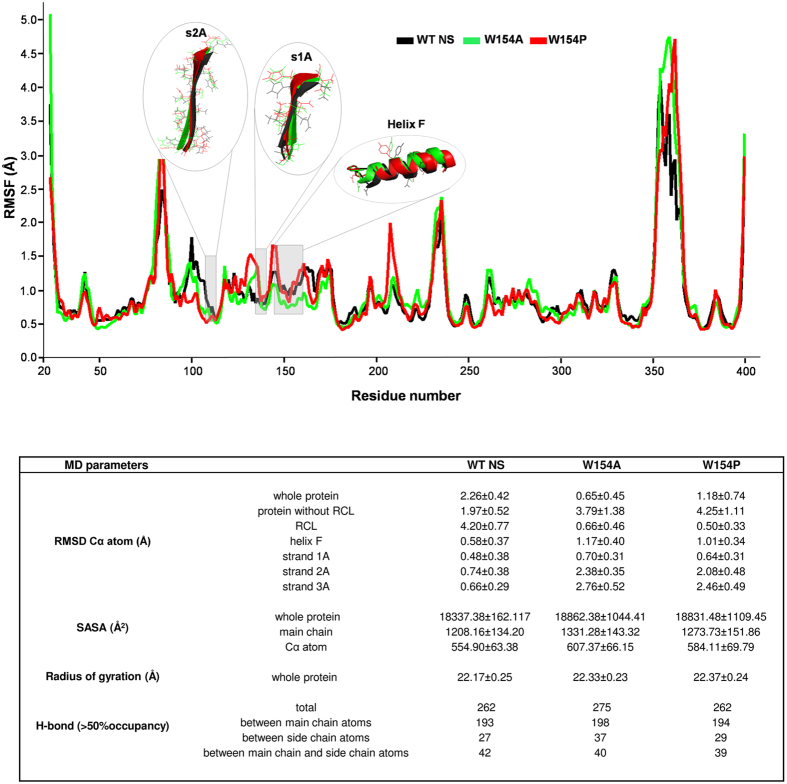
MD simulation. Root Mean Square Fluctuations (RMSF) of all residues of WT NS (black line), W154A (green line) and W154P (red line) were calculated after energy minimization essentially as described in material and methods. The highlighted region represents the larger observed fluctuations in helix F, strands 1A and 2A in W154A and W154P compared to WT NS. Table showing Root Mean Square Displacement (RMSD) at maximum stationary value, Solvent Accessible Surface Area (SASA), radius of gyration and hydrogen bond with more than 50% occupancy.

**Figure 3:**
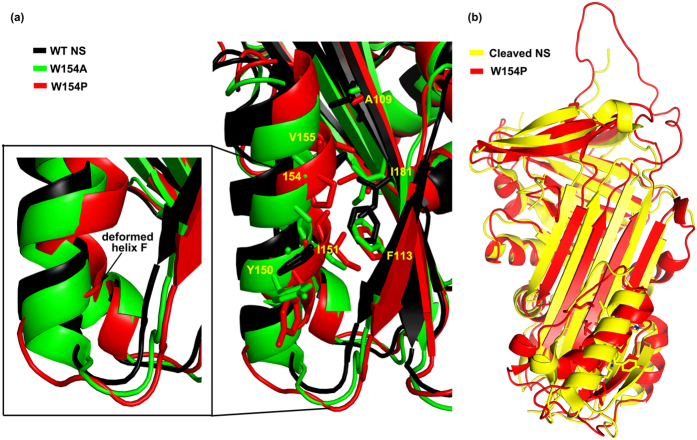
Structural superposition of modeled 154 variants and cleaved NS. (**a**) Superposition of WT NS (black), W154A (green) and W154P (red) after 40 ns simulation. (**b**) Superposition of the cleaved NS (yellow) and W154P (red) showing the shortening of helix F at its N-terminal end and movement of the hydrophobic residues. The images were generated using PyMOL^48^.

**Figure 4:**
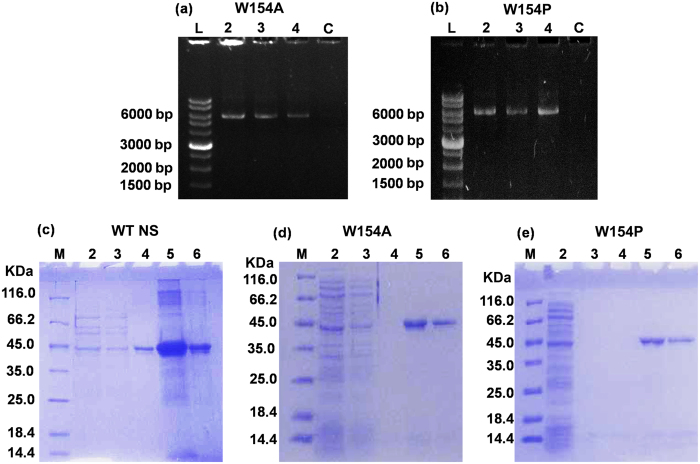
Site-directed mutagenesis and purification. 0.8% agarose gel image of PCR amplification of (**a**) W154A and (**b**) W154P using plasmid containing WT NS gene as a template. 12% SDS-PAGE image of Ni-NTA purification of (**c**) WT NS, (**d**) W154A and (**e**) W154P Lane M, 2, 3, 4, 5 and 6 corresponds to protein marker, washing with high salt buffer, washing with 50 ml buffer A, washing with 20 mM Tris-HCl [pH 8.0], elution with 300 mM imidazole in buffer A and elution with 600 mM imidazole in buffer A, respectively. Fractions of Lane 5 and 6 were pooled, buffer exchanged and concentrated to run on Gel-filtration column to remove impurities.

**Figure 5:**
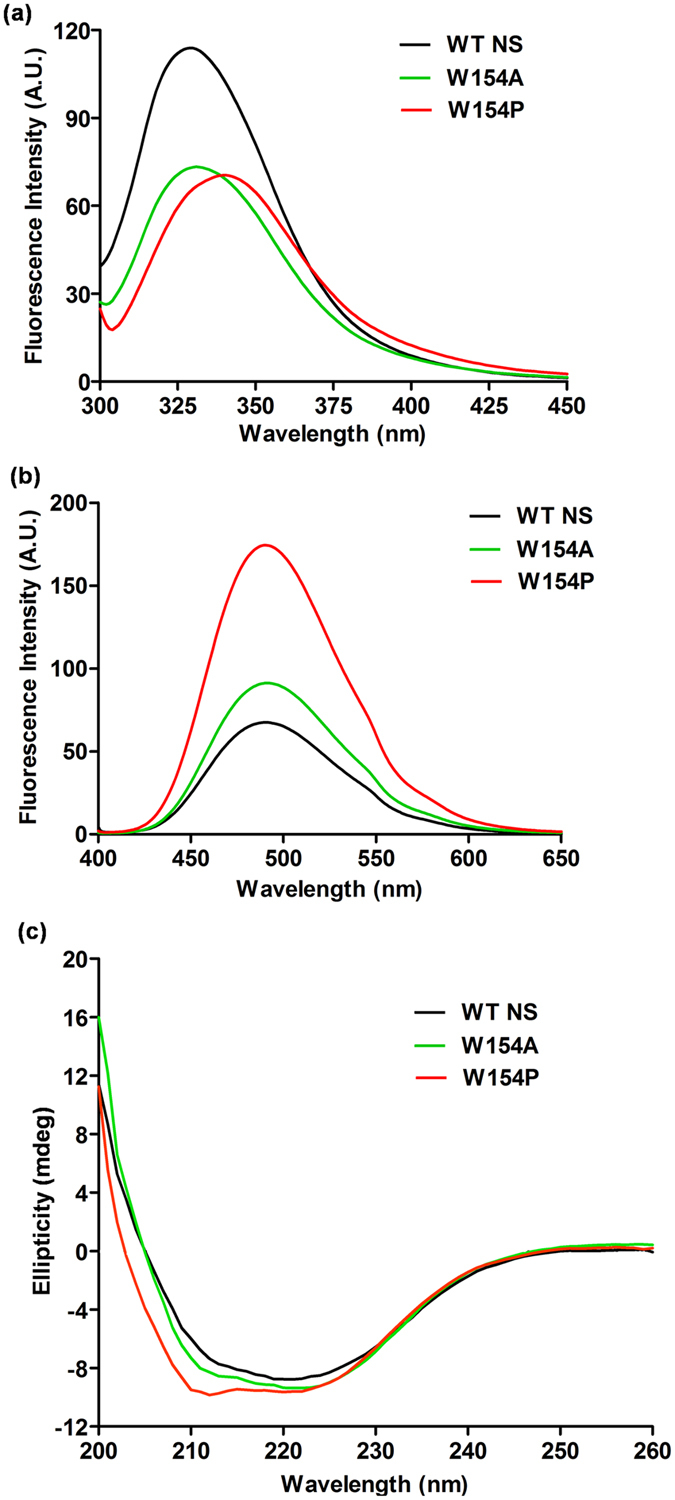
Biophysical characterizations. (**a**) Fluorescence emission spectra, (**b**) bis-ANS fluorescence, (**c**) far-UV CD spectra of WT NS (black line), W154A (green line) and W154P (red line). Each spectrum is an average of at least three individual experiments.

We have compared the MD trajectories and RMSF plots of WT NS and the W154 variants to show that there are marked changes in helix F region, strands 1A and 2A (Fig. 2). The W154A variant exhibited reduced mobility in the helix F region and strand 2A and increased mobility in strand 1A and RCL. Furthermore, a structural superposition of the modeled structures suggested that W154P helix F was deformed at N-terminal end resulting in more flexibility in the loop connecting helix F to strand 1A (Fig. 3a). Consequently fluctuations in helix F of W154P and strand 1A are more as compared to W154A. The *in silico* observations implied that perturbation in the hydrophobic patch surrounding W154 affects strand 1A and strand 2A of β-sheet A whereas strand 3A remains unperturbed. The results agree with a previous study that investigated the role of strand 3A by engineering a disulfide bond with helix F, and showed that the bond didn’t affect inhibition mechanism but it influenced the rate of polymerisation^21^.

**Figure 6:**
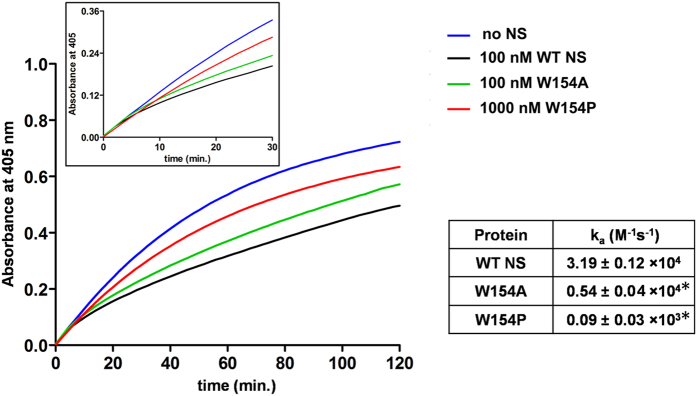
Inhibition assay. Progressive curve showing hydrolysis of chromogenic substrate T2943 from Sigma CH_3_SO_2_-D-HHT-G-R-pNA.AcOH by tPA at 25 °C in the presence of 0 NS (blue line), 100 nM WT NS (black line), 100 nM W154A (green line) and 1000 nM W154P (red line). Inset: enlarged view of first 30 min of reaction. Table showing k_a_ of WT NS, W154A and W154P, with ± standard deviation of at least three independent experiments. *The data for k_a_ was found to be statistically significant with P < 0.001 for both W154A and W154P. One way ANOVA was used for determining statistical significance and P value was determined using t-test.

**Figure 7:**
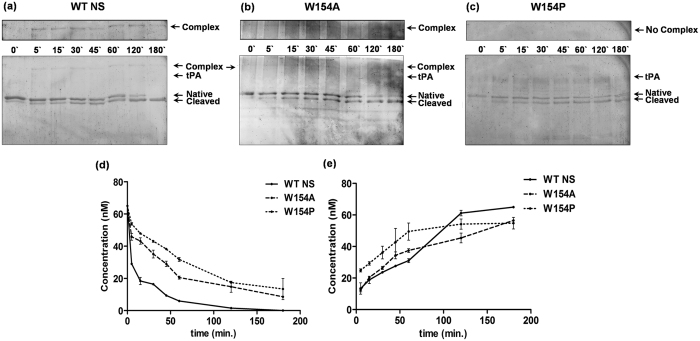
Complex formation analysis of WT NS and 154 variants. Complex formation of (**a**) WT NS (**b**) W154A (**c**) W154P with tPA in 50 mM HEPES buffer [pH 7.4] at 25 °C. Aliquots of reaction were taken at different time intervals from 0 to 180 min. Samples were run on 12% SDS-PAGE. Each lane contains 65 nM NS and 13 nM tPA. The arrow indicates the different species: complex, tPA, native NS and cleaved NS on SDS-PAGE. The band densities from SDS-PAGE (**d**) WT NS and (**e**) cleaved NS (WT NS (solid lines), W154A (dashed lines) and W154P (dotted lines)).

**Figure 8:**
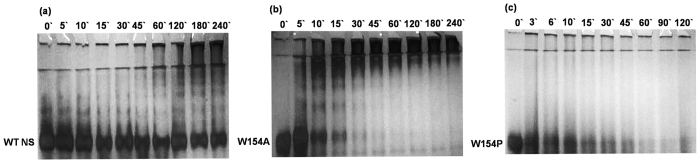
Polymerisation assay. 10% non-denaturing PAGE of (**a**) WT NS, (**b**) W154A, (**c**) W154P showing polymerisation upon incubation at different time intervals at 45 °C.

The analysis of the W154P variant demonstrates absence of NS-tPA complex formation and appearance of cleaved NS (Fig. 7c). Previous studies have shown that NS-tPA complex are unstable and can dissociate in minutes unlike other serpin-protease complexes that can have half life of upto weeks^30^,^36^,^41^,^42^. A similar type of increase in substrate pathway has also been demonstrated by binding a ligand specific for helix F^43^ in PAI-1. W154A forms a complex with tPA and showed inhibition (Figs 6 and 7b). We observed that most of the hydrophobic interactions with W154 are with indole ring of the tryptophan (Fig. 1a) and hypothesized that, W154A variant will reduce the number of contacts with residues originating from strands 1A, 2A and 3A of the β-sheet A, but will maintain the integrity of helix F as the interaction with V155 and Y150 will be maintained (Figs 1a, 2 and 3a). Increased polymerisation propensity was observed for W154A variant based on the faster disappearance of band of NS and appearance of polymers as compared to very slow polymer transition observed in the WT NS (Fig. 8). W154P native band disappeared at 45 min with only large molecular weight aggregates, indicating that this variant is in a partially unfolded conformation and a W154P type variant might affect the integrity of helix F (Figs 2 and 3). Indeed, the complete deletion in helix F of PAI-1 showed complete loss in inhibitory activity and purely substrate behavior^44^.

In the present study we report that a conserved hydrophobic patch surrounding W154 may play a crucial role in the control of helix F dynamics. We hypothesize that partial deformation of helix F and relaxation in the loop connecting strand 1A will shift helix F in a synchronized manner with strands 1A and 2A. This coordinated movement is important for not allowing a gap to form between the strands of β-sheet A before insertion of the strand 4A, any variant which alters this organization will increase the polymerisation propensity. This hypothesis needs to be tested more vigorously using variants of conserved hydrophobic residues identified in this study.

## Methods

### Molecular Dynamics (MD) Simulations

Initial coordinates of WT NS (PDB ID: 3FGQ)^29^ were obtained from Brookhaven Protein Data Bank RCSB-PDB (www.rcsb.org). The missing loop coordinates were generated using Swiss-Model web server by keeping 3FGQ as a template^31^. MD simulations were carried out on WT, W154A and W154P models of NS under similar environmental conditions using AMBER_14 running on Tesla K20c CUDA workstation. For an explicit solvent environment, both the proteins were immersed into a virtual box having distance between protein to box wall equal to 0.5 Å. Each box is then solvated with TIP3P water molecules^45^ (18,000 and 19,000 TIP3P box water molecules respectively) before subjecting the proteins to energy minimization. This is followed by addition of counter ions (17 Na^+^) for charge neutralization of the minimized system, followed by re-minimization of the ionized solvated systems. Equilibration of the minimized systems was performed at a low temperature of 100 K and gradually heating up to 300 K by maintaining constant volume temperature with no pressure coupling. Long-range electrostatic interactions were evaluated by the Particle Mesh Ewald (PME) method, where Van der Waals and Coulomb interactions were truncated using a switch function within 1 nm cutoff. SHAKE algorithm was applied to constraint all the covalent bonds with H atoms, using non-bonded cutoff set to 12 Å. Thermal bath of 300 K was coupled to 1.0 Atm pressure in each of the subjected simulations and the coupling parameters for both pressure and temperature were set as 1 ps. Using Integration step of 2 fs, a final 40 ns production run simulation was carried out for all the minimized and equilibrated systems. The resulting trajectories were analyzed using AmberTools 14 to determine the dynamics of W154 dependent interactions and associated mutational consequences.

### Sub cloning and site-directed mutagenesis

The plasmid pQE81L containing human NS gene was kindly provided by Prof. David A. Lomas, University of Cambridge, Cambridge, UK. It was sub cloned with restriction sites *NheI* and *XhoI* into the pET28b (Novagen) expression vector encoding N-terminal poly-His-tag and human NS protein was confirmed by MALDI-TOF analysis. The W154P mutant was constructed by polymerase chain reaction (PCR) based site-directed mutagenesis with mutagenic primers forward: 5′AACTACATCAATAAGCCGGTGGAGAATAAC3′, reverse: 5′GTTATTCTCCACCGGCTTATTGATGTAGTT3′; and mutagenic primers for W154A mutant were: forward: 5′AACTACATCAATAAGGCGGTGGAGAATAAC3′, reverse: 5′GTTATTCTCCACCGCCTTATTGATGTAGTT3′ using plasmid containing NS gene as a template. PCR product was transformed in *E. coli* DH5α (Novagen) cells following *Dpn1* treatment. Both the mutations were confirmed by DNA sequencing of complete gene (Supplementary Fig. S3).

### Expression and purification of recombinant WT and mutant proteins

The WT NS, W154A and W154P constructs were transformed into *E. coli* BL21/DE3 cells (Novagen) for the expression of recombinant proteins. 1% of overnight bacterial culture was inoculated to 4 liter of Luria Bertani (LB) broth (Hi-Media) containing 50 μg/ml kanamycin and incubated at 37 °C at 200 rpm. The cells were grown to an optical density at 600 nm (OD_600_) of 0.6 and cell culture was induced with 0.5 mM isopropyl-β-D-thiogalactopyranoside and kept for 16 hr while shaking at 20 °C. The cells were harvested by centrifugation at 7000 rpm for 10 min at 4 °C and cell pellets were stored at −80 °C for later use. The cell pellets were resuspended in lysis buffer comprising of 30 mM Tris-HCl [pH 8.0], 150 mM NaCl, 5 mM imidazole, 0.1% Triton X-100 and 5% glycerol along with 100 μM phenylmethylsulfonyl fluoride and 0.1 mg/ml lysozyme (Sigma-Aldrich, St. Louis MO, USA). After incubating on ice for 30 min, the cell suspension was sonicated on ice at 40% of amplitude for 2 min with impulse of 10 sec interspersed with an interval of 20 sec. NS protein was purified by two-step chromatography. Initially, the crude cellular extract obtained after centrifugation at 15000 rpm at 4 °C was applied onto Ni-NTA Sepharose column (GE Healthcare) and protein bound beads were washed with 10 ml of high salt buffer [30 mM Tris-HCl [pH 8.0], 500 mM NaCl, 10 mM imidazole and 5% glycerol]. Further washing with 50 ml of buffer A [30 mM Tris-HCl [pH 8.0], 150 mM NaCl, 10 mM imidazole, 5 mM β-mercaptoethanol (βME) and 5% glycerol] followed by washing with 30 ml of 20 mM Tris-HCl [pH 8.0] were carried out. Finally, protein was eluted using 300 mM and 600 mM imidazole in buffer A at 4 °C. Eluted fractions were run on 12% SDS-PAGE to check the presence of NS. The elution fractions containing protein were pooled, concentrated using Amicon ultracentrifugal filters (Millipore) and loaded on to HiLoad 16/60 Superdex 200 column (GE Healthcare) at flow rate of 1 ml/min for further purification. The column was pre-equilibrated with running buffer; 30 mM Tris-HCl [pH 8.0], 150 mM NaCl, and 5% glycerol before loading the protein. Monomeric purified proteins were used for carrying out the experiments.

### Fluorescence measurement

Structural differences between WT NS and 154 variants were monitored by fluorescence measurement study using JASCO FP-6300 spectrofluorometer at 25 °C. The tryptophan fluorescence was detected upon excitation at 295 nm and emission spectra were recorded from 300–450 nm. The excitation and emission slit widths were set at 5 nm. All experiments were performed in 20 mM sodium phosphate buffer [pH 7.4] with 2 μM of protein samples. Each spectrum represents an average of three individual scans and was corrected for the contribution of the blank solution.

### 4,4′-Bis-1-anilino naphthalene 8-sulfonate (bis-ANS) fluorescence spectra

Bis-ANS is a widely used extrinsic fluorescence probe to check the surface hydrophobicity of proteins^46^. Post mutation changes in the overall surface hydrophobicity of NS were assessed by fluorescence spectra of bis-ANS (Sigma-Aldrich, St. Louis MO, USA). For this experiment protein samples (2 μM) were incubated with bis-ANS in a molar ratio of 1:5 at 25 °C for 1 hr in dark. Fluorescence measurements were performed using a JASCO FP-6300 spectrofluorometer. Bis-ANS emission spectra were detected using an excitation wavelength of 390 nm, and spectra were recorded from 400–650 nm^46^. Each spectrum represents the mean of three individual experiments, and were baseline corrected by subtracting with appropriate buffer and protein blanks.

### Circular Dichroism studies

Circular Dichroism (CD) spectra were recorded on Chirascan^TM^Plus CD Spectrometer from Applied Photophysics with peltier temperature control. Far-UV CD spectra were recorded at protein concentration of 0.2 mg/ml in the range of 260–200 nm wavelength at rate of 1 nm step. Far-UV CD spectrum was performed at least thrice where each reading represents the average of 10 scans taken at 25 °C. All CD spectra were baseline-corrected by substracting the buffer spectrum and smoothed. CD experiments were performed in 20 mM sodium phosphate buffer [pH 7.4].

### Inhibition assay

To determine the kinetic parameter of NS, inhibition assays were performed under pseudo-first order rate reaction, using the progressive curve method as described previously^8^,^35^. Briefly, inhibition assay was initiated with the addition of 100 nM tPA (>85% sctPA, Molecular Innovations, USA) to the mixture of 1 mM chromogenic substrate (T2943 [CH_3_SO_2_-D-HHT-G-R-pNA.AcOH] from Sigma-Aldrich, St. Louis MO, USA) and varying concentration of WT NS (100 nM, 200 nM, 400 nM, 600 nM and 1000 nM) in kinetic buffer 50 mM HEPES [pH 7.4], 150 mM NaCl, and 0.1% Tween-20 at 25 °C. Progress of reaction was recorded with release of the product with absorbance at 405 nm as a function of time. Inhibition assay of W154A was performed at the same concentration as that for WT NS, but W154P was performed at a higher concentration (0.6 μM, 1 μM, 2 μM and 3 μM) as negligible inhibition was observed at lower concentration. Values of pseudo-first order rate constant (k_obs_),


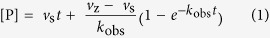



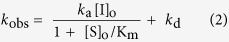


initial velocity (v_z_) and steady-state velocity (v_s_) were determined upon fitting the progressive curve in equation (1). Further, association rate constant (k_a_) was calculated using equation (2).

### Complex formation assay

NS and tPA are known to form covalent complex^2^. In order to determine the progress of complex formation, 625 nM of NS was incubated with 125 nM of sctPA at 25 °C in kinetic buffer 50 mM HEPES [pH 7.4], 150 mM NaCl, and 0.1% Tween-20. Aliquots (25 μl) of the sample were removed at time intervals of 5, 15, 30, 45, 60,120 and 180 min of incubation and mixed immediately with preheated SDS-loading buffer at 95 °C to stop the reaction. Further, samples were frozen at −80 °C till the completion of the experiment. Time 0 min samples were made by the addition of NS to denatured sctPA (heating at 95 °C in SDS loading buffer). Samples were taken out of −80 °C and heated for 5 min before loading on 12% SDS-PAGE. Gels were stained with fluorescent nimble juice speedy protein gel stain from Genedirex (Taiwan) and visualized on UV transilluminator.

### Polymerisation assay

Polymerisation assays were assessed by incubating protein samples at 1 mg/ml at 45 °C in 20 mM HEPES buffer [pH 7.4]. Aliquots (3 μl) were taken at different time intervals and mixed with 1:1 loading buffer 250 mM Tris-HCl [pH 6.8], 50% glycerol and 0.5% bromophenol blue and snap frozen in liquid nitrogen and stored at −80 °C. Samples were taken out from −80 °C after completion of the experiment and run on 10% non-denaturing PAGE. Gels were stained with silver staining kit from Thermo Fisher Scientific for visualization.

## Additional Information

**How to cite this article:** Ali, M. F. *et al*. A hydrophobic patch surrounding Trp154 in human neuroserpin controls the helix F dynamics with implications in inhibition and aggregation. *Sci. Rep.* **7**, 42987; doi: 10.1038/srep42987 (2017).

**Publisher's note:** Springer Nature remains neutral with regard to jurisdictional claims in published maps and institutional affiliations.

## Supplementary Material

Supplementary Information
