# Supplementary material for: A hydrophobic patch surrounding Trp154 in human neuroserpin controls the helix F dynamics with implications in inhibition and aggregation
Source: Sci Rep. 2017 Feb 23;7:42987. doi: 10.1038/srep42987 (PMC5322333; doi:10.1038/srep42987)
Supplement: Supplementary Information [file srep42987-s1.pdf]

Supplementary Information

A hydrophobic patch surrounding Trp154 in human neuroserpin controls the helix F dynamics with implications in inhibition and aggregation

Mohammad Farhan Ali[1], Abhinav Kaushik[2], Charu Kapil[1], Dinesh Gupta[2] and Mohamad Aman Jairajpuri[1]*

*Correspondance to [email: mjairajpuri@jmi.ac.in]

[1]Protein Conformation and Enzymology Lab, Department of Biosciences, Jamia Millia Islamia (A Central University), New Delhi-110025, India

[2]Translational Bioinformatics Group, International Center for Genetic Engineering and Biotechnology, Aruna Asaf Ali Marg, New Delhi-110067, India

Supplementary Figure S1. Multiple sequence alignment (MSA). MSA shows a conserved Trp at position 154 in helix F of NS (indicated by a yellow star mark) (a) orthologs (b) among serpin superfamily members. MSA was carried out using Clustal Omega[1] and secondary structure with MSA was done using Espript 3.0[2].

Supplementary Figure S2. Root Mean Square Displacements (RMSD). Backbone RMSD as a function of time WT NS (black), W154A (green) and W154P (red) in 40 ns MD simulation.

Supplementary Figure S3. Electropherograms. Indicating (a) W154A and (b) W154P showing mutated nucleotide leading to expression of alanine and proline at position 154 in W154A and W154P variants respectively.

Supplementary Figure S4. GdmCl induced denaturation. Fluorescence emission spectra of (a) WT NS, (b) W154A and (c) W154P at different GdmCl concentration. (d) Fluorescence emission maxima (λ_{max}) of WT NS, W154A and W154P at different GdmCl concentration.

References

1. McWilliam, H. *et al.* Analysis Tool Web Services from the EMBL-EBI. *Nucleic Acids Res* **41**, W597-60 (2013).
2. Gouet, P., Robert, X. & Courcelle, E. ESPript/ENDscript: Extracting and rendering sequence and 3D information from atomic structures of proteins. *Nucleic Acids Res* **31**, 3320-3323 (2003).

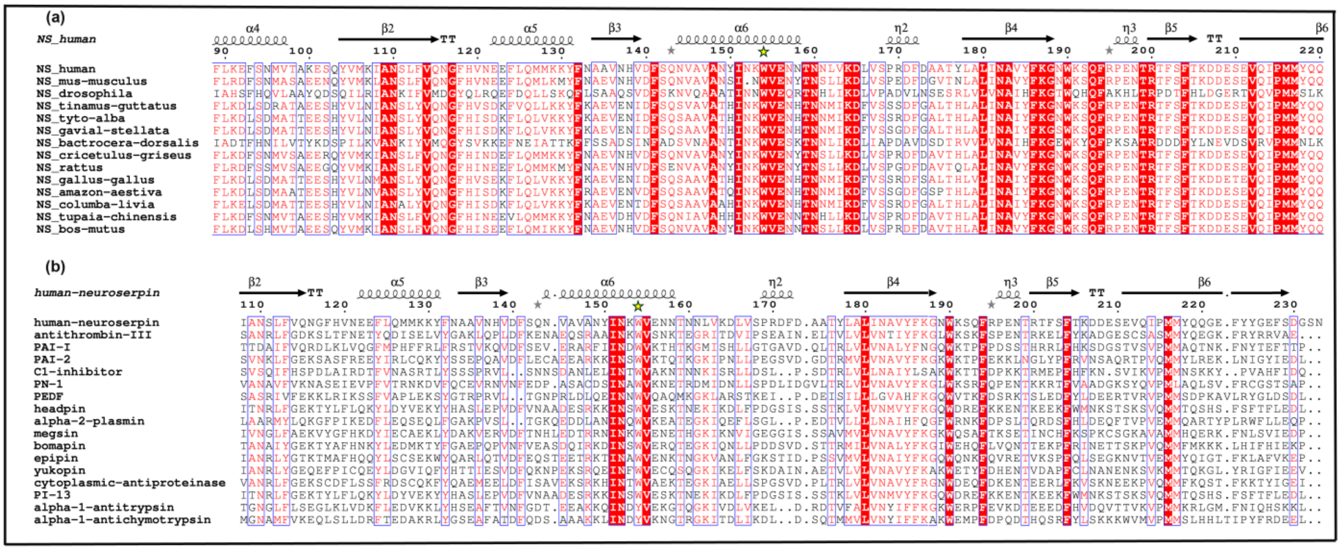

Supplimentary Fig. S1

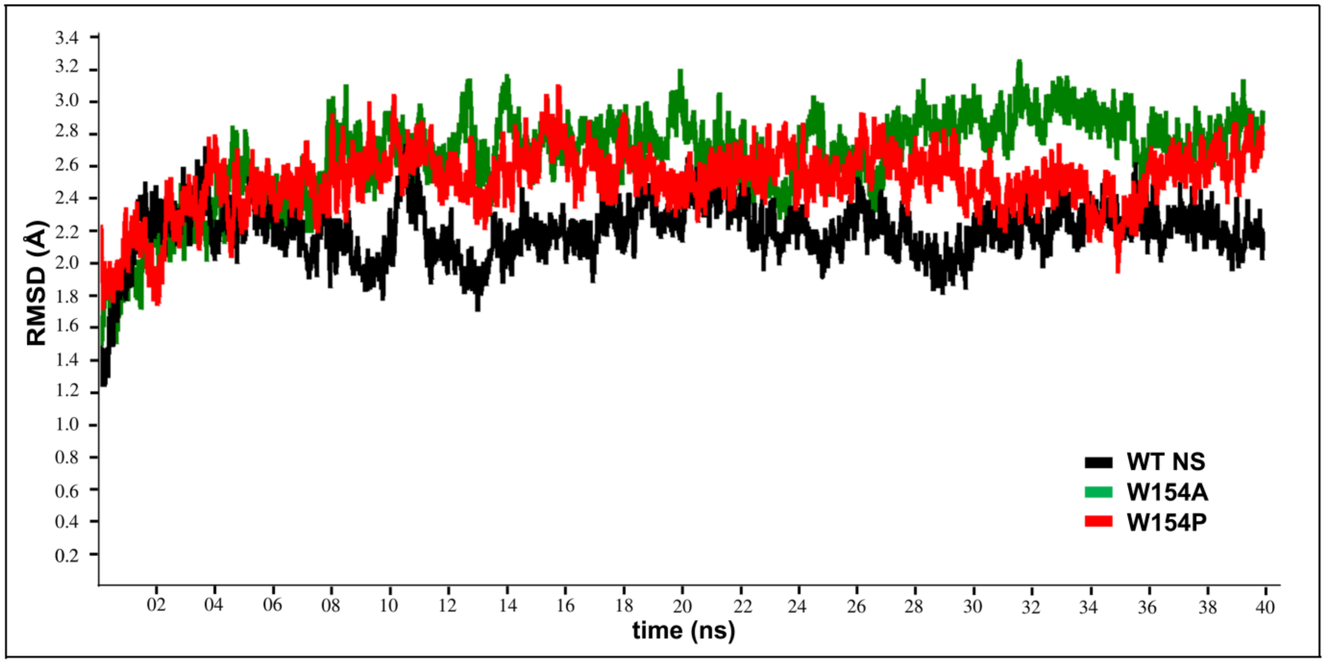

Supplimentary Fig. S2

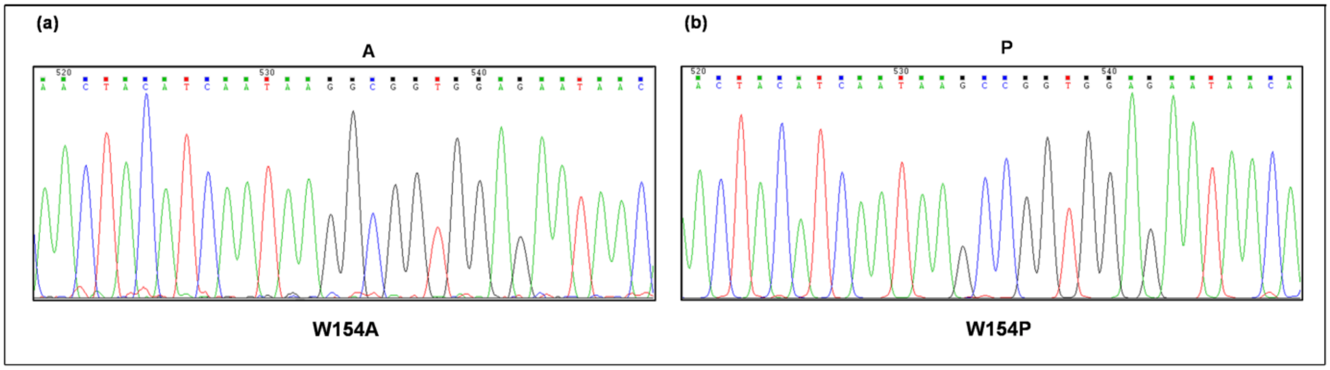

Supplimentary Fig. S3

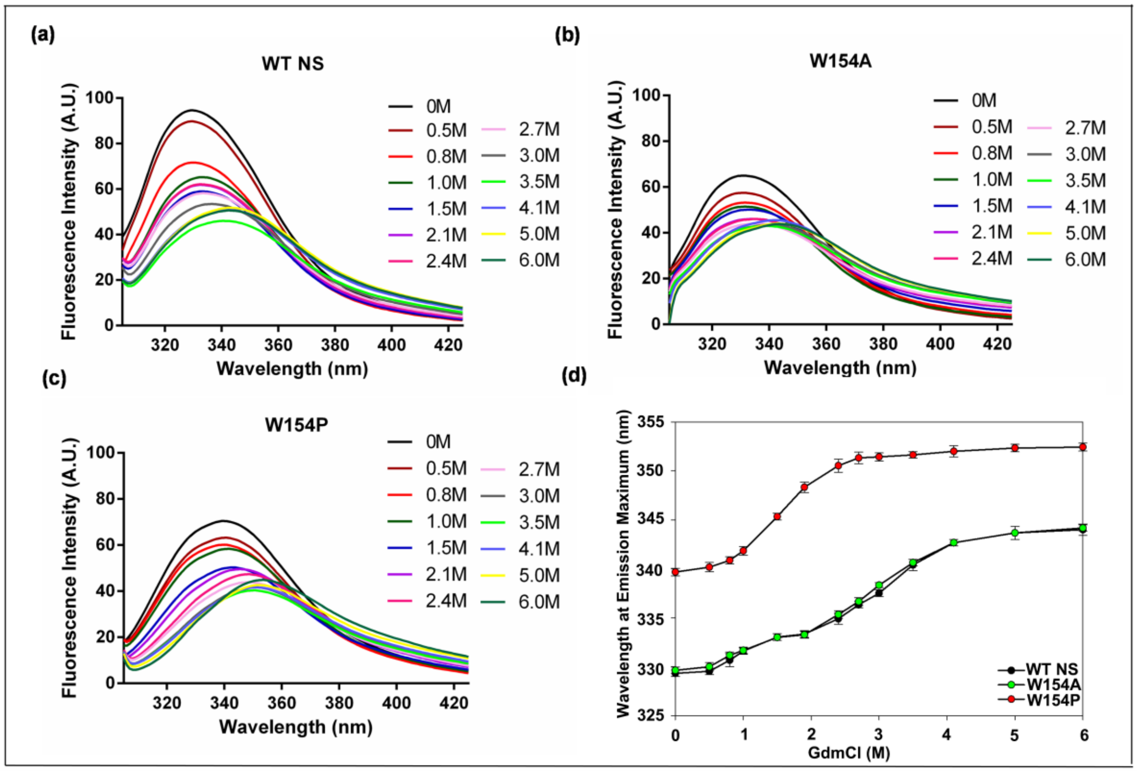

Supplimentary Fig. S4
